# Development of a visual detection method of porcine deltacoronavirus using loop-mediated isothermal amplification

**DOI:** 10.3389/fmicb.2024.1465923

**Published:** 2024-09-16

**Authors:** Renfeng Li, Wenyan Cao, Jiakang Yuan, Linyue Li, Yanlin Zhou, Fangyu Wang, Ziliang Wang, Xiangqin Tian

**Affiliations:** ^1^College of Animal Science and Veterinary Medicine, Henan Institute of Science and Technology, Xinxiang, China; ^2^Sanquan College of Xinxiang Medical University, Xinxiang, China; ^3^Key Laboratory of Animal Immunology of the Ministry of Agriculture, Henan Provincial Key Laboratory of Animal Immunology, Henan Academy of Agricultural Sciences, Zhengzhou, China; ^4^Henan Key Laboratory of Medical Tissue Regeneration, Xinxiang Medical University, Xinxiang, China

**Keywords:** porcine, deltacoronavirus, loop-mediated isothermal amplification, visualization, detection

## Abstract

The emergence of porcine deltacoronavirus (PDCoV) presents a significant threat to both human and animal health due to its ability to cause highly contagious enteric diseases. This underscores the crucial need for timely and accurate diagnosis to facilitate effective epidemiological investigation and clinical management. This research aimed to establish a visual detection method based on reverse transcription loop-mediated isothermal amplification (RT-LAMP) for PDCoV testing. In this study, six pairs of primers were designed according to the conserved sequences of PDCoV ORF1a/b genes. The primer sets and parameters that affect LAMP reaction were optimized. The visual RT-LAMP method was developed by incorporating methyl red into the optimized reaction system, it exclusively detected PDCoV without cross-reactivity with other viruses and the detection limits for PDCoV could reach 10 copies/μL. In comparison with RT-PCR for testing 132 clinical samples, the relative specificity and sensitivity of the visual RT-LAMP were found to be 99.2 and 100%, respectively, with a concordance rate of 99.2% and a kappa value of 0.959, indicating that the visual RT-LAMP is a reliable method for the application of PDCoV detection in clinical samples.

## Introduction

PDCoV is a newly identified swine enteropathogenic coronavirus, belonging to the family *Coronaviridae*, genus *Deltacoronavirus*, and it is an enveloped, single-stranded RNA virus with a genome size of approximately 25.4 kb (Liu and Wang, 2021). Pigs at various stages of development are susceptible, with suckling piglets aged 5–15 days being particularly susceptible. Infection can lead to acute watery diarrhea, vomiting, dehydration, lethargy and reduced appetite in piglets, and in severe cases piglets may die (Wang et al., 2019).

The initial detection of PDCoV was reported in an extensive molecular epidemiological study undertaken in Hong Kong in 2012, with a focus on mammals and birds (Woo et al., 2012). However, it was not until early 2014 that an outbreak of swine diarrhea due to PDCoV infection was witnessed on a pig farm in Ohio, United States. Analysis of fecal and intestinal samples from the affected pigs revealed that the pathogen strain possessed a high genome sequence homology of 99% to the PDCoV strain previously identified in Hong Kong, China (Wang et al., 2014). This pathogenic strain proliferated swiftly across multiple states, and its pathogenicity was subsequently established. Further on, the virus was discerned in piglet fecal samples from various other countries, such as Canada, South Korea, Japan, Thailand, Vietnam, Mexico, and Laos (Marthaler et al., 2014; Duan, 2021).

In recent years, there has been a discernible upward trajectory in the occurrences of PDCoV outbreaks (Shan et al., 2024). The escalating infection rates pose a substantial menace to the swine industry not only in China but also on a global scale (Jung et al., 2016). Moreover, the prevalence of PDCoV has extended beyond swine, encompassing poultry and other mammals as well (Jung et al., 2017; Boley et al., 2020; Wang et al., 2023). Notably, researchers have unveiled the presence of PDCoV in plasma samples obtained from three Haitian children afflicted with acute undifferentiated febrile illness (Lednicky et al., 2021). This discovery underscores the potential for PDCoV to undergo interspecies transmission.

The absence of commercially available vaccines against PDCoV necessitates the development of rapid and precise diagnostic tools for effective prevention and control measures. Currently employed methods for PDCoV detection include reverse transcription-polymerase chain reaction (RT-PCR), real-time RT-PCR, enzyme-linked immunosorbent assay (ELISA), virus isolation, and virus neutralization, etc. (Wang et al., 2022; Chen et al., 2023a; Hou et al., 2023; Zhao et al., 2023). However, these methods pose challenges for on-site use in resource-limited settings due to their time-consuming, labor-intensive, and expensive nature, compounded by the scarcity of highly skilled personnel and the need for costly specialized equipment.

LAMP is a molecular biology technique that facilitates the rapid and efficient amplification of nucleic acid sequences under isothermal conditions (Hassan et al., 2022; Park, 2022). Results can be read simply by observing fluorescence or turbidity visually in the reaction tube with no additional process (Sema et al., 2015). Currently, LAMP has gained prominence due to its simplicity, speed, and applicability in various fields, including diagnostics, pathogen detection, and molecular biology research (Garg et al., 2022; Sharma et al., 2022). Its ability to work under relatively simple conditions without the need for complex thermal cycling equipment makes LAMP an attractive tool for point-of-care testing in resource-limited settings (Chen et al., 2023b).

In the context of eradicating PDCoV in China, it is crucial to have a diagnostic method that is rapid, user-friendly, and highly specific for PDCoV prevention and control. While the potential of LAMP as a laboratory tool is recognized, its diagnostic efficacy for PDCoV in China has not been assessed. This study aims to establish a visual RT-LAMP assay and evaluate its diagnostic performance in comparison to RT-PCR for PDCoV detection.

## Materials and methods

### Viruses, plasmid, and samples

Porcine epidemic diarrhea virus (PEDV), PDCoV, transmissible gastroenteritis virus (TGEV), porcine reproductive and respiratory syndrome virus (PRRSV), and standard plasmid harboring the PDCoV ORF1a/b gene were obtained from the Key Laboratory of Animal Viral Diseases Prevention and Control at Henan Institute of Science and Technology. A total of 132 clinical fecal samples of pigs were collected from different pig farms of Xinxiang region in Henan province, China.

### Primer design

Employing Primer Premier 5.0 software, six primer sets of RT-LAMP were designed to target the highly conserved ORF1a/b gene of PDCoV (GenBank accession number: MN942260.1) for highly specific detection. Each primer set comprises an outer pair (F3/B3) and an inner pair (FIP/BIP). In addition, the PDDB primer set was applied in RT-PCR and the target product was 359 bp. All primers were synthesized by Shanghai Sangon Biotechnology Co., Ltd. The specific sequences of each primer set are presented in Table 1.

**Table 1:** Primers used in the visual RT-LAMP assay.

Primer sets^a^		Sequences (5′ → 3′)
PDORF1	F3	ACCACCACCTCTGCAACT
	B3	GCCTCACACACCAGATTACG
	FIP	GCCTTGGTGGCGTAGAGGTTATAGCTGCTGAAGAGGGTCA
	BIP	GCCTGGCGTGCAGGTTCTTAGGGTTGAGCTCCAAAGACAT
PDORF2	F3	CGCACTCTATTCCTGGAA
	B3	CCCACAACAACTTCATGTT
	FIP	ATGGGTTTCATAAAGAGACTGTTCACAGGTGTCATTCATAGAGACG
	BIP	GTCAGTCTATGGTTAGGGATGCGGTGTATCCTACTACAACACGC
PDORF3	F3	CTAAAGCTACTTTTGTCATTGAC
	B3	ATACAACTGTGAACCGCC
	FIP	CTTGTTGTAAGAGGCCAGATACTGGAAAAGTACGTGTTGCTTAAAGAC
	BIP	TTCTGGTACTGCTTCTGATAAGGAACAAAGCCTTGGCAAGAA
PDORF4	F3	GAATATGACCCAATACAACCAA
	B3	CCTATGATAGGTAGCTACAACAT
	FIP	TGCACCTTTTCCACTCTGAGGAGGTCTACTGCATGAAGTG
	BIP	CCTACCGGTCCTGCCATTAAAAGTGTCATAGCCATTAACTGT
PDORF5	F3	GCCTCTCTTCCAGGATTC
	B3	TCCGGAAAGTGGTCTGTT
	FIP	GTGTTTCGTTGATTTGAGCATTAGTAGCTTTTCATAAGCTTTAACTGC
	BIP	GCTTAACCAACTAGTGGCTCCAGCATCTGCAAGTGTATCG
PDORF6	F3	TCTGGACATTTTGGATCTTTG
	B3	AACAAATTTACAGGTAAGATCGA
	FIP	TGCCTTACATAGTGCTCTACCCAGATTCGTAAACCCAAATTCATCC
	BIP	GTCTTTGTCAGTGCTTCGCTTTTGGACAACCTTATTGTTGAGTCAA
PDDB	PDDF	ATTCTGCTTTGGCTGCTC
	PDDR	TCCTGTGGCGGATTTC

a FIP/BIP: inner primer set; F3/B3: outer primer set; PDDF/PDDR: primer set for RT-PCR amplification.

### RT-LAMP

The RT-LAMP reaction system was established according to the manufacturer’s instructions (Bst 2.0 WarmStart DNA polymerase, NEB, United Kingdom). The reaction mixture consisted of 2.5 μL of 10× isothermal amplification buffer, 1.5 μL of MgSO_4_ (100 mmol/L), 3.5 μL of dNTPs Mix (10 mmol/L), 1 μL of Bst 2.0 WarmStart DNA polymerase (8,000 U/mL), 1 μL each of F3/B3 primers (5 μmol/L), 1 μL each of FIP/BIP primers (40 μmol/L), 1 μL of standard plasmid as the template, and ddH_2_O to a total volume of 25 μL. Using the established LAMP system, six primer sets were screened forsubsequent visual RT-LAMP assay.

To optimize the LAMP reaction conditions, the parameters including reaction time (10 min, 20 min, 30 min, 40 min, 50 min, 60 min, 70 min, and 80 min), reaction temperature (54°C, 56°C, 58°C, 60°C, 62°C, 64°C, 66°C, and 68°C), inner primer concentration (8 μmol/L, 16 μmol/L, 24 μmol/L, 32 μmol/L, 40 μmol/L, 48 μmol/L, 56 μmol/L, and 64 μmol/L), outer primer concentration (1 μmol/L, 2 μmol/L, 3 μmol/L, 4 μmol/L, 5 μmol/L, 6 μmol/L, 7 μmol/L, and 8 μmol/L), Bst enzyme concentration (1,000 U/mL, 2,000 U/mL, 4,000 U/mL, 6,000 U/mL, 8,000 U/mL, 10,000 U/mL, 12,000 U/mL, and 14,000 U/mL), Mg^2+^ concentration (0 mmol/L, 20 mmol/L, 40 mmol/L, 60 mmol/L, 80 mmol/L, 100 mmol/L, 120 mmol/L, and 140 mmol/L), dNTPs concentration (1–8 represent 4 mmol/L, 5.5 mmol/L, 7 mmol/L, 8.5 mmol/L, 10 mmol/L, 11.5 mmol/L, 13 mmol/L, and 14.5 mmol/L), and buffer concentration (dilution of 0.4×, 0.6×, 0.8×, 1.0×, 1.2×, 1.4×, 1.6×, and 1.8×) were evaluated. The optimal conditions were determined based on agarose gel electrophoresis results.

### Development of visual RT-LAMP assay

Using the optimized reaction system, LAMP amplification was performed with 1,000-fold diluted standard plasmid as the template (ddH_2_O as the negative control). Methyl red (2.6 mmol/L) was added to the optimized system to visualize the amplification results. The detection was confirmed by observing color changes before and after the reaction, and the reaction solution was also analyzed by agarose gel electrophoresis.

### Evaluation of visual RT-LAMP for PDCoV detection performance

The optimized visual RT-LAMP reaction system was used to individually detect PEDV, PDCoV, PRRSV, and TGEV. Negative (ddH_2_O) and positive (1,000-fold diluted standard plasmid) control were included. The specificity was evaluated by observing color change in the tubes and agarose gel electrophoresis.

To assess the sensitivity of the visual RT-LAMP assay, standard plasmid was serially diluted in ddH_2_O to concentrations ranging from 10^8^ to 1 copy/μL. Visual RT-LAMP reactions were performed using these dilutions, and the results were analyzed by agarose gel electrophoresis and color change simultaneously.

### Detection of PDCoV in clinical samples

The fecal samples were resuspended in a quintupling volume of sterile phosphate buffered saline (PBS) and subjected to centrifugation for deletion of debris, and the resulting supernatant was filtered through a 0.45 μm filter. Subsequently, viral RNA was extracted from the filtered supernatant using a Viral RNA Mini Kit (QIAGEN, Germany). Finally, the RNA was reverse transcribed into cDNA using a PrimeScriptII 1st-strand cDNA synthesis kit (TakaRa, Japan), and then subjected to RT-PCR and the visual RT-LAMP assay. The RT-PCR program was as follows: initial denaturing at 95°C for 5 min; followed by 33 cycles at 95°C for 30 s, 5°C for 40 s, and 72°C for 1 min; and a final extension at 72°C for 10 min. The concordance rate between the two methods was evaluated by Cohen’s kappa value.

## Results

### Primer screening

**Figure 1:**
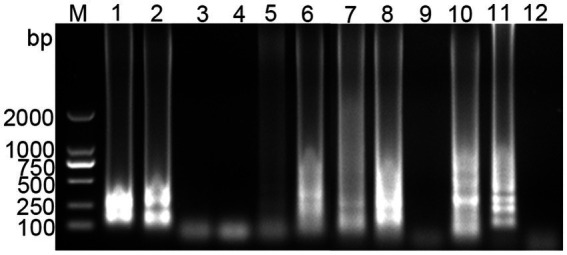
Screening results of PDCoV RT-LAMP primer sets. M for 2,000 bp DNA marker, lanes 1 and 2 represent positive and negative amplification results of primer sets 1, respectively; lanes 3 and 4 for primer sets 2; lanes 5 and 6 for primer sets 3; lanes 7 and 8 for primer sets 4; lanes 9 and 10 for primer sets 5; lanes 11 and 12 for primer sets 6.

Using the standard plasmid as the template, we conducted LAMP with six different sets of primers. The amplification results were analyzed through agarose gel electrophoresis. As illustrated in Figure 1, primer set 6 yielded the most intense positive band, while the negative control showed no bands. Consequently, primer set 6 was chosen for further experiments.

### Optimization of RT-LAMP reaction system

**Figure 2:**
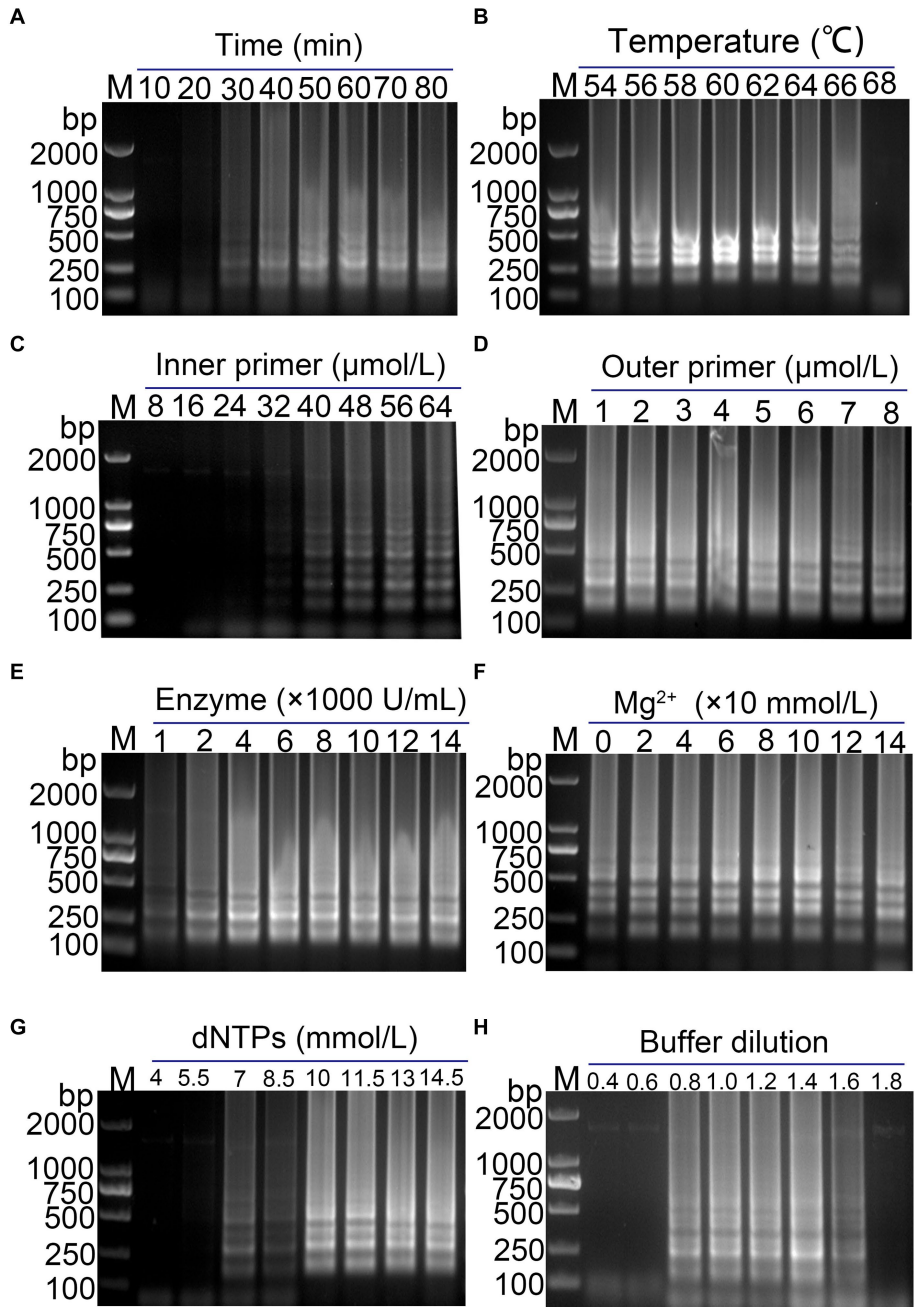
Optimization of the PDCoV RT-LAMP reaction system. **(A)** Reaction time; **(B)** reaction temperature; **(C)** inner primer concentration; **(D)** outer primer concentration; **(E)** Bst enzyme concentration; **(F)** Mg^2+^ concentration; **(G)** dNTPs concentration; **(H)** Buffer concentration. The final optimized conditions are as follows: reaction 50 min **(A)** at 60°C **(B)**, the concentrations of inner primer and outer primer were 48 μmol/L **(C)** and 5 μmol/L **(D)**, respectively. The optimal concentrations of Bst enzyme, Mg^2+^, dNTPs and buffer were found to be 4,000 U/mL **(E)**, 100 mmol/L **(F)**, 10 mmol/L **(G)**, and 1.4× **(H)**, respectively.

Optimization of RT-LAMP reaction system was performed to determine the optimal conditions for amplification. As shown in Figure 2, the optimal reaction time and temperature were determined to be 50 min (Figure 2A) and 60°C (Figure 2B), respectively. The optimal inner primer concentration was found to be 48 μmol/L (Figure 2C). The outer primer concentration had a minimal impact on the LAMP reaction, and a concentration of 5 μmol/L was selected for the reaction (Figure 2D). The optimal enzyme concentration was determined to be 4,000 U/mL (Figure 2E), with the band brightness reaching its brightest at this concentration. The optimal concentrations of Mg^2+^, dNTPs and buffer were found to be 100 mmol/L (Figure 2F), 10 mmol/L (Figure 2G), and 1.4× (Figure 2H), respectively. The final optimized reaction system was established, and the reaction was performed at 60°C for 50 min, followed by inactivation at 80°C for 10 min.

### Establishment of visual RT-LAMP assay

**Figure 3:**
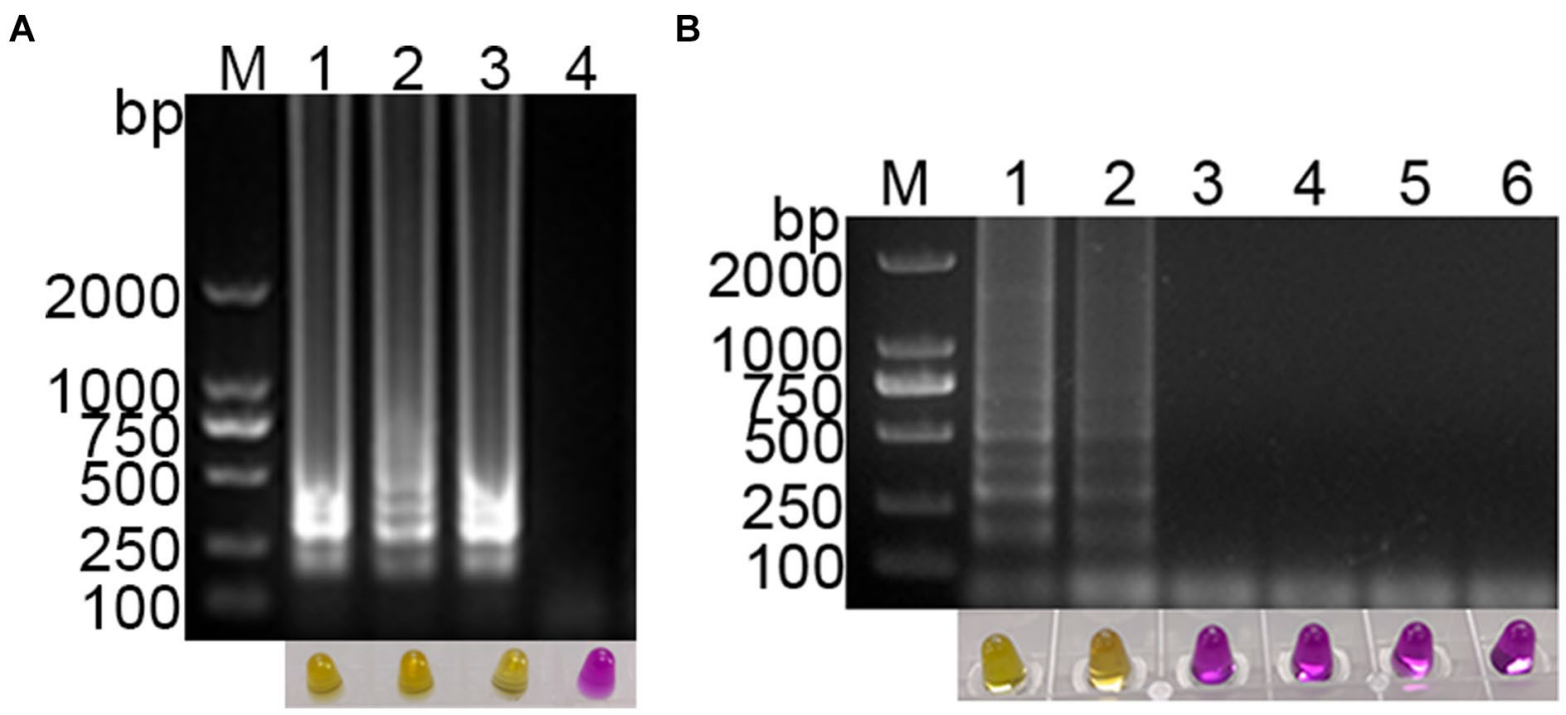
Establishment of visual PDCoV RT-LAMP assay. **(A)** The results of RT-LAMP gel electrophoresis and visual RT-LAMP with methyl red indicator; **(B)** specificity evaluation of the visual RT-LAMP assay.

The feasibility of using methyl red as a visual indicator for the LAMP reaction was evaluated. A 1,000-fold dilution of standard plasmid was used as the template, and ddH_2_O was used as the negative control. Methyl red (2.6 mmol/L) was added to the optimized RT-LAMP reaction system, and the LAMP reaction was conducted. The results showed clear gradient bands in the positive amplification gel electrophoresis, while no bands were observed in the negative reaction gel electrophoresis (Figure 3A). Simultaneously, the color in the positive reaction tube changed from purple red to yellow, while the color in the negative reaction tube remained purple red (Figure 3A). This indicates that methyl red was a suitable indicator for the visual LAMP reaction, facilitating rapid and straightforward detection of the target nucleic acid of PDCoV.

### Specificity of visual RT-LAMP assay

The specificity of the established PDCoV visual RT-LAMP assay was evaluated using cDNA from PDCoV, PEDV, PRRSV, and TGEV as templates, ddH_2_O as the negative control, and a 1,000-fold dilution of standard plasmid as the positive control. The results showed that PEDV, PRRSV, and TGEV, as well as the negative control, exhibited no positive bands in gel electrophoresis, and the color in the tubes remained purple red. In contrast, the standard plasmid and PDCoV cDNA displayed gradient bands in gel electrophoresis, with the color in the tubes changing from purple red to yellow (Figure 3B). These findings suggest that the established PDCoV visual RT-LAMP detection method can specifically detect PDCoV without cross-reacting with other porcine viruses.

### Sensitivity of visual RT-LAMP assay

**Figure 4:**
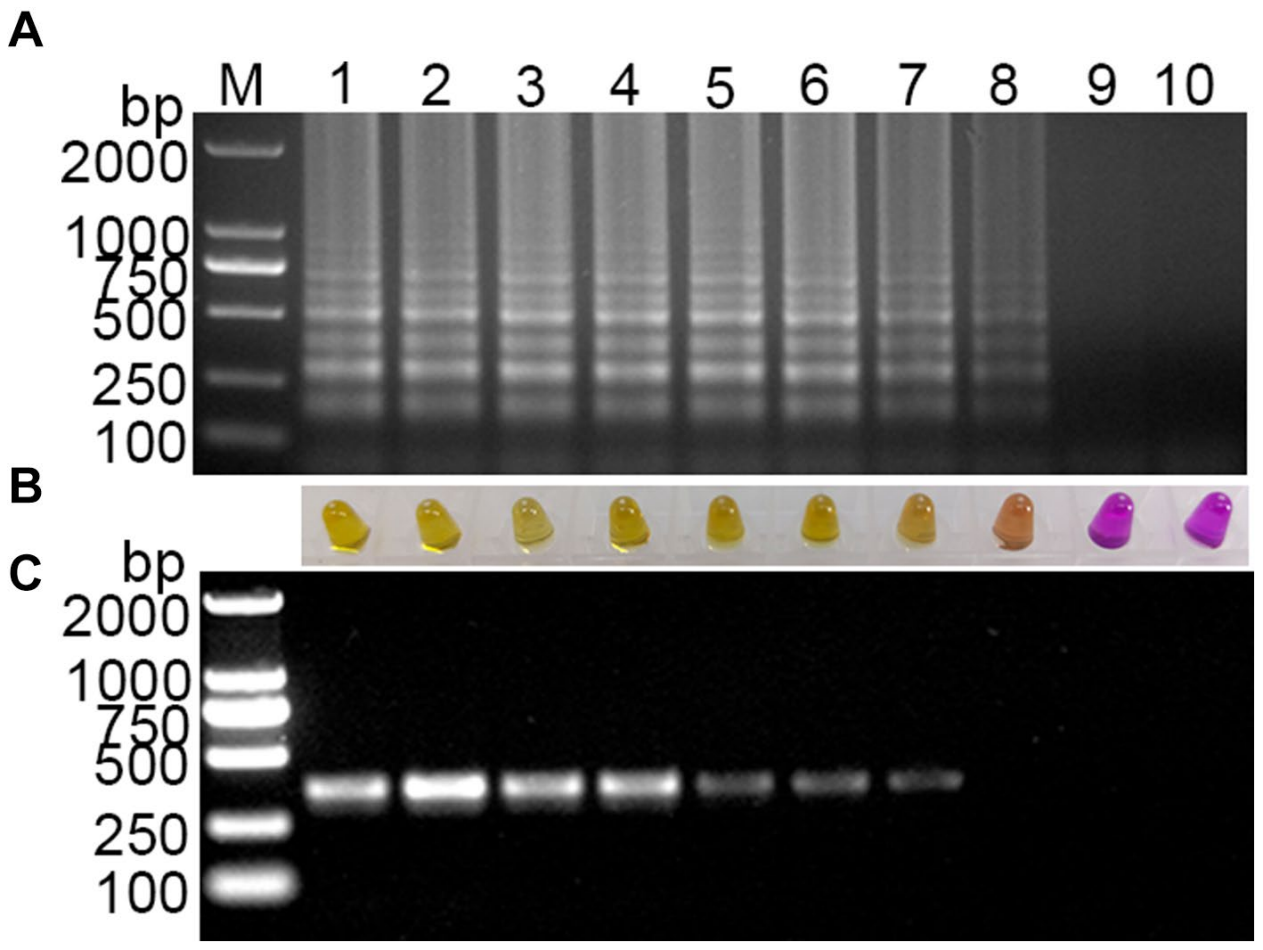
Sensitivity evaluation of the visual RT-LAMP assay. **(A)** The results of RT-LAMP gel electrophoresis; **(B)** the results of RT-LAMP with methyl red indicator; **(C)** the results of RT-PCR gel electrophoresis: M for 2,000 bp DNA marker, lanes 1–6 represent DNA marker, lanes 1–9 represent 10^8^–1 copies/μL standard plasmid; lane 10 represents negative control.

Using a template with concentrations ranging from 10^8^ to 1 copies/μL of standard plasmid, the established visual RT-LAMP and the RT-PCR method were employed simultaneously for PDCoV detection. The visual RT-LAMP showed that even at 10 copies/μL, bands were detectable, and the color in the tubes changed to yellow. In comparison, the RT-PCR results showed that the standard plasmid could be detected at 10^2^ copies/μL (Figure 4). The detection limit of the visual RT-LAMP was determined to be 10 copies/μL, with a sensitivity one order of magnitude higher than RT-PCR.

### Comparison between the visual RT-LAMP and RT-PCR for detection of clinical samples

A total of 132 clinical pig fecal samples were tested using the visual RT-LAMP and RT-PCR. As shown in Table 2, RT-PCR detected 14 PDCoV-positive and 118 PDCoV-negative samples, resulting in a positivity rate of 10.6% (14/132). The visual RT-LAMP detected 15 PDCoV-positive and 117 PDCoV-negative samples, with a positivity rate of 11.4% (15/132). Compared with RT-PCR, the specificity and sensitivity of the visual RT-LAMP were 99.2 and 100%, respectively. There was excellent agreement between the results obtained by the two methods (kappa = 0.959). These findings indicate that the visual RT-LAMP assay developed in this study possesses good clinical detection capabilities and can be employed for PDCoV clinical testing.

**Table 2:** Comparison between the visual RT-LAMP and RT-PCR for testing PDCoV.

		RT-PCR		Total
		Positive	Negative	
Visual RT-LAMP	Positive	14	1	15
	Negative	0	117	117
	Total	14	118	132

A total of 132 samples were tested by the visual RT-LAMP and RT-PCR. Relative to RT-PCR, diagnostic specificity of the visual RT-LAMP: 117/118 × 100% = 99.2%; diagnostic sensitivity of the visual RT-LAMP: 14/14 × 100% = 100%; Po = (14 + 117)/132 = 0.992; Pe = [(14/132) × (15/132) + (118/132) × (117/132)] = 0.804, kappa = (Po − Pe)/(1 − Pe) = 0.188/0.196 = 0.959.

## Discussion

In recent years, isothermal amplification techniques have gained popularity in veterinary diagnostics. Various methods for identifying the newly emerged PDCoV have been developed using these techniques, such as single-tube one-step LAMP (Zhang et al., 2017), cleaved probe-based reverse transcription LAMP (CP-RT-LAMP) (Shen et al., 2022), recombinase polymerase amplification (RPA) combined with a lateral flow dipstick (LFD-RPA) (Gao et al., 2020), and probe-based reverse transcription RPA (RT-RPA) assay (Ma et al., 2019). Compared to traditional PCR, LAMP offers higher sensitivity, specificity, and simplicity, eliminating the need for temperature cycling to achieve amplification. However, LAMP is more prone to false positives due to aerosol contamination and primer complexity. To mitigate this, it is advisable to keep the reaction covered as much as possible during and after the reaction (Soroka et al., 2021). If opening the reaction is necessary after completion, the sample loading area, amplification area, and nucleic acid electrophoresis area should be separated, or a sealant can be used to prevent aerosol contamination (Dhama et al., 2014). Additionally, primers are a critical component of LAMP amplification, playing a key role in ensuring the specificity, efficiency, and sensitivity of the DNA amplification process. Multiple primers need to be designed and optimized in terms of a range of factors, including concentration, location of nucleotide pairs, and distance between DNA regions (Soroka et al., 2021). In this study, we chose the ORF1a/b gene of PDCoV as the target. The gene fragment of ORF1a/b accounts for about 2/3 of the PDCoV genome, which is longer than the commonly used M and N genes, making it easier to obtain suitable primer combinations and reducing the workload of primer design and screening.

The LAMP reaction generates substantial amounts of amplified product in the form of double-stranded DNA, along with a byproduct, magnesium pyrophosphate (Wong et al., 2018). A widely used approach involves inducing a nucleic acid-dependent color change by incorporating pH-sensitive dyes directly into the LAMP reaction. This integration is crucial for minimizing the risk of contamination and enabling clear color differentiation between positive and negative samples (Tanner et al., 2015). However, potential limitations of using pH-sensitive dyes include the assay’s potential inability to generate sufficient pH variation and the requirement for a weakly buffered solution, which limits their use in the LAMP reaction. An alternative method uses the hydroxy naphthol blue (HNB) metal indicator dye, which has shown improved sensitivity compared to pH-sensitive dyes in weakly buffered solutions. This is due to the synergistic effect of LAMP’s byproducts (pyrophosphate-PPi^4−^ and hydrogen-H^+^ ions). However, these methods may produce false-positive results due to nonspecific amplification (Grammatikos et al., 2023). In the present study, methyl red was selected as the indicator to integrate into the RT-LAMP system. This choice enabled a noticeable shift in color from purple-red to yellow, facilitating result interpretation through visual inspection and rendering it more appropriate for on-site rapid testing in comparison to commonly utilized LAMP indicators such as HNB (Kim et al., 2021), SYBR Green- I fluorescence dye (Li et al., 2022), and calcein (Zhao and Feng, 2019). Furthermore, methyl red can be added to the reaction system of LAMP in advance, avoiding the need to open the reaction tube after completion and reducing the risk of aerosol contamination.

Our newly developed visual RT-LAMP assay can identify PDCoV at 10 copies/μL within 50 min. This method is more sensitive than the traditional RT-PCR technique for clinical PDCoV test (PDCoV positivity rates of 11.4 and 10.6%, respectively) and is similar to a previous RT-LAMP test that used SYBR Green I dye (Zhang et al., 2017). Additionally, this new assay takes less time than qRT-PCR and SYBR Green I-based RT-LAMP methods. Nevertheless, it still encounters challenges such as the inability for multiplex detection, direct RNA amplification, and achieving “sample-answer” detection. With the ongoing advancement of microfluidic technology in the detection field, researchers have successfully integrated microfluidic chips with LAMP technology to develop more convenient and rapid nucleic acid detection methods (Rodriguez et al., 2016; Shang et al., 2020; El-Tholoth et al., 2021). These methods leverage the advantages of LAMP and microfluidic systems, providing new directions for the rapid detection of PDCoV. One promising approach is to adopt paper-based microfluidics, which offers a low-cost and portable platform for LAMP assays. This could enable the development of new detection methods for PDCoV that are more accessible and user-friendly.

One limitation of our study is that the visual RT-LAMP assay was not tested across a broader range of environmental conditions and sample types beyond the clinical fecal samples from pigs. While our assay demonstrated high sensitivity and specificity under laboratory conditions, its performance in diverse field settings, including potential interference from environmental contaminants, remains untested. Future studies should aim to validate the assay’s robustness and accuracy in various environmental conditions to ensure its applicability for on-site testing in different geographical regions and under varying environmental factors.

## Conclusion

In this study, a visual detection method of RT-LAMP was developed for PDCoV testing. The optimized visual RT-LAMP assay exhibited high sensitivity and specificity, providing a rapid, intuitive tool for the early detection of PDCoV in clinical settings, contributing to improved pig disease management, outbreak control, and surveillance of PDCoV infections.

## Data Availability

The raw data supporting the conclusions of this article will be made available by the authors, without undue reservation.
